# Diagnosing Tuberculosis: What Do New Technologies Allow Us to (Not) Do?

**DOI:** 10.1159/000525142

**Published:** 2022-06-27

**Authors:** Shima M. Abdulgader, Anna O. Okunola, Gcobisa Ndlangalavu, Byron W.P. Reeve, Brian W. Allwood, Coenraad F.N. Koegelenberg, Rob M. Warren, Grant Theron

**Affiliations:** ^a^DSI-NRF Centre of Excellence for Biomedical Tuberculosis Research, South African Medical Research Council Centre for Tuberculosis Research, Division of Molecular Biology and Human Genetics, Faculty of Medicine and Health Sciences, Stellenbosch University, Cape Town, South Africa; ^b^Division of Pulmonology, Department of Medicine, Tygerberg Hospital, Stellenbosch University, Cape Town, South Africa

**Keywords:** Tuberculosis, Non-invasive testing, Rapid tests, Diagnosis

## Abstract

New tuberculosis (TB) diagnostics are at a crossroads: their development, evaluation, and implementation is severely damaged by resource diversion due to COVID-19. Yet several technologies, especially those with potential for non-invasive non-sputum-based testing, hold promise for efficiently triaging and rapidly confirming TB near point-of-care. Such tests are, however, progressing through the pipeline slowly and will take years to reach patients and health workers. Compellingly, such tests will create new opportunities for difficult-to-diagnose populations, including primary care attendees (all-comers in high burden settings irrespective of reason for presentation) and community members (with early stage disease or risk factors like HIV), many of whom cannot easily produce sputum. Critically, all upcoming technologies have limitations that implementers and health workers need to be cognizant of to ensure optimal deployment without undermining confidence in a technology that still offers improvements over the status quo. In this state-of-the-art review, we critically appraise such technologies for active pulmonary TB diagnosis. We highlight strengths, limitations, outstanding research questions, and how current and future tests could be used in the presence of these limitations and uncertainties. Among triage tests, CRP (for which commercial near point-of-care devices exist) and computer-aided detection software with digital chest X-ray hold promise, together with late-stage blood-based assays that detect host and/or microbial biomarkers; however, aside from a handful of prototypes, the latter category has a shortage of promising late-stage alternatives. Furthermore, positive results from new triage tests may have utility in people without TB; however, their utility for informing diagnostic pathways for other diseases is under-researched (most sick people tested for TB do not have TB). For confirmatory tests, few true point-of-care options will be available soon; however, combining novel approaches like tongue swabs with established tests like Ultra have short-term promise but first require optimizations to specimen collection and processing procedures. Concerningly, no technologies yet have compelling evidence of meeting the World Health Organization optimal target product profile performance criteria, especially for important operational criteria crucial for field deployment. This is alarming as the target product profile criteria are themselves almost a decade old and require urgent revision, especially to cater for technologies made prominent by the COVID-19 diagnostic response (e.g., at-home testing and connectivity solutions). Throughout the review, we underscore the importance of how target populations and settings affect test performance and how the criteria by which these tests should be judged vary by use case, including in active case finding. Lastly, we advocate for health workers and researchers to themselves be vocal proponents of the uptake of both new tests and those − already available tests that remain suboptimally utilized.

## Introduction

Despite efforts to eradicate tuberculosis (TB), a large gap remains between the estimated 10 million cases and the 7.1 million cases newly diagnosed each year [1]; crippling elimination efforts. This has prompted increased emphasis on the identification of cases as early as possible, including in communities, to curtail transmission [2]. However, the fight against TB has recently been jeopardized: COVID-19 has undone years of progress, with TB cases rising globally for the first time in more than a decade, largely due to the compromised ability of patients to readily access health facilities and rapid tests [3]. This impact on TB diagnostic services coupled with the overall slow technological progress of test development is alarming when juxtaposed against the rapid development and deployment of novel and accurate point-of-care (POC) SARS-CoV-2 tests.

TB tests broadly fall across two non-mutually exclusive categories. First, in the triage category, are tests that identify who, in a pool of at-risk people, may have disease (such individuals may be pre- or asymptomatic) and require further TB investigations (a positive triage result increases the probability of TB and triage-negative patients should typically not require further investigation for TB). Such tests can be used to efficiently allocate patients to receive critical yet expensive confirmatory tests (confirmatory tests can be done without triage yet this is not cost-effective or affordable) [4]. Triage is also important because confirmatory test capacity is often unevenly distributed (some settings have very limited capacity and in others, capacity is underutilized [5]). Hence, triage tests can increase not just efficiency but, if scalable, also the variety and volume of at-risk individuals referred for confirmatory testing.

Most current triage activity in national TB programmes involves symptom screening, which is subjective, misses asymptomatic or minimally symptomatic cases, and is vulnerable to poor quality-of-care [6]. An approach complementary to syndromic screening is chest X-ray (CXR). Traditional CXR requires significant infrastructure and human readers, which precludes wide usage in primary care; however, portable low-dose imaging hardware and computer-aided detection (CAD) software have advanced rapidly [7] and outperform experienced human readers [8, 9, 10], Critically, triage testing should not just be strengthened at a facility level (where cases, often undetected, are already present [11, 12]) but also at a community level to detect TB before it passively enters the health system (if at all); thereby breaking the transmission cycle. Triage test specificity is the key determinant of effectiveness (and cost savings) as it determines the proportion of at-risk individuals who do not need further testing [4]. Importantly, community-based triage testing is, diagnostically, inherently more challenging than facility-based testing as pretest probabilities of TB are lower (reducing positive predictive value), and patients have earlier-stage disease, which is more likely to be paucibacillary and harder to detect.

The second TB test use case is confirmatory testing, which is used post-triage to identify people that have TB. Such tests, therefore, provide a positive result to justify starting a patient on treatment (or conversely, if negative, justify not starting treatment). Currently, confirmatory tests are primarily done at distant district centralized laboratories using a nucleic acid amplification test (NAAT) like Xpert MTB/RIF Ultra [13] (Ultra; Cepheid), Truenat MTB (Truenat) [14], or culture methods like Mycobacteria Growth Indicator Tube 960. In addition to improving decentralization (which has important benefits vs. centralized testing [15]) and rapidity, a major challenge that needs to be overcome for a confirmatory testing revolution to occur is to show highly accurate non-sputum confirmatory diagnoses are possible in routine primary care settings without specimens needing to leave clinics. One key reason non-sputum testing is itself needed is because people living with HIV (PLHIV) and those not (yet) symptomatic, as well as children and patients with extrapulmonary TB, often cannot produce high-quality sputum. Importantly, a triage test including symptoms can alone be used to start treatment in the absence of a conclusive confirmatory test result. This empiric treatment is dependent on clinical factors that affect pretest probability and logistical and practical considerations [16].

**Fig. 1:**
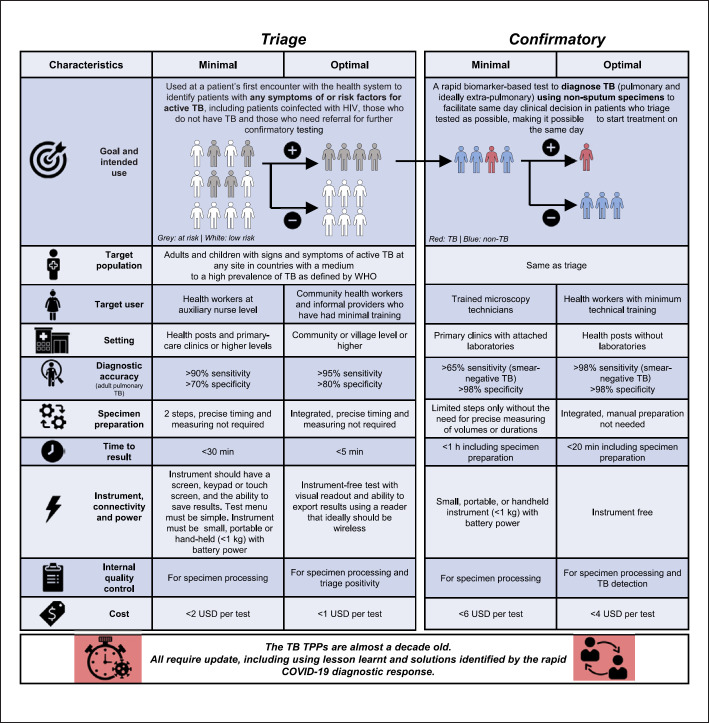
Selected minimal and optimal characteristics of the WHO target product profiles for TB triage and confirmatory tests. Current and future technologies should be benchmarked against these criteria, which are needed to advance the diagnostic status quo. Although serious attempts to meet the TPP criteria are underway, these criteria are aspirational, and, if a particular test or technology falls short in some domains it should not be discarded. Importantly, these criteria are almost a decade old, and, although they remain key to guide developers, they urgently require update to include lessons learnt from the rapid development and scale-up of SARS-CoV-2 detection technologies. Adapted from the WHO [17].

A true POC triage or confirmatory test that enables same-day result reporting in a single clinical encounter without requiring technical expertise remains elusive. Despite being almost a decade old, the World Health Organization (WHO) target product profiles (TPPs) are the only global benchmarks that researchers and test developers have for performance (Fig. 1), and, despite the TPPs' age, no presently available diagnostics meet optimal triage or confirmatory criteria [17]. The availability of the TPP specifications to developers and researchers for a long period without any commercial products being able to meet these criteria raises the question of whether a universal test capable of meeting these benchmarks in a broad range of settings is possible, given the wide spectra of TB disease, human and mycobacterial genetic diversity, and co-morbidities. In other words, different patient- or setting-niches may require different tests, thresholds, and/or test combinations to approach optimal TPP criteria. Lastly, further indicative of the TPPs' age, TPPs for recently recharacterized early forms of active TB (e.g., subclinical disease) do not exist, and the TPPs are ill-fitting for new digital technologies like artificial intelligence (AI)-based specimen-free approaches (e.g., sounds, imaging) and other solutions like connectivity brought to the foreground by the COVID-19 response.

It is noteworthy, however, that several currently or imminently available tests perform comparatively well in certain subpopulations and settings. These groups are unfortunately often a minority of patients from an epidemiological population perspective; however, such patients are still clinically important to target. For example, the urinary Alere Determine TB LAM lateral flow (AlereLAM) test is the only TB test with evidence from a randomized controlled trial of a mortality benefit and meets TPP criteria in HIV-positive inpatients [18]. Second, the recently developed Xpert MTB Host Response (MTB-HR) test may meet minimum performance criteria yet more data are needed [19]. Despite these few bright spots, however, this overwhelming shortage of TPP-compliant POC tests in the context of a long-established and worsening global TB pandemic is ironic because, in contrast, SARS-CoV-2 already has rapid, accurate, and low-cost tests already available. This is due to the unprecedented level of resources brought to bear on the COVID-19 pandemic, which in the space of a few years is in already orders of magnitude greater than that made available throughout the entire history of TB.

**Fig. 2:**
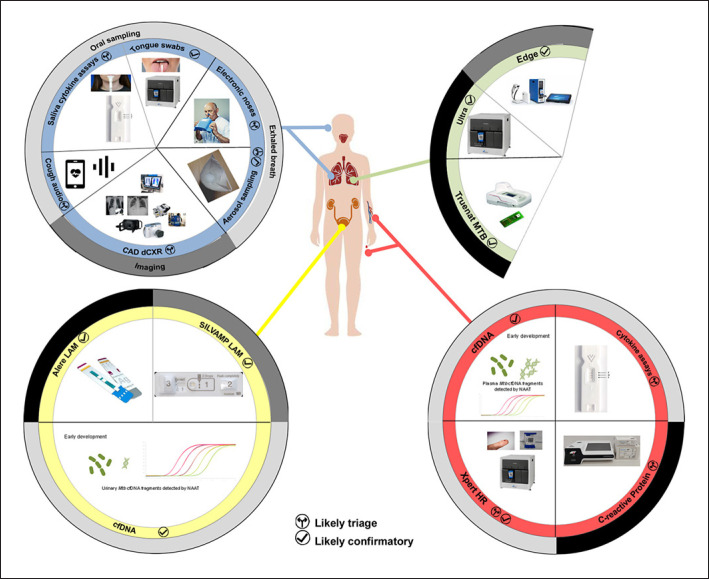
Selected current and future triage and confirmatory tests according to specimen type (green, sputum; red, blood; yellow, urine; blue, other). Black shading on the outer ring indicates WHO-endorsed; grey indicates on pathway to the WHO review; light grey indicates still at early developmental stages. Image sources include https://www.fujifilm.com/products/medical/digital_radiography/fdr_xair/, https://www.delft.care/cad4tb/, https://www.fujifilm.com/products/medical/data/Lunit_INSIGHT_CXR_Medical_White_Paper.pdf, http://www.stoptb.org/wg/new_diagnostics/assets/documents/TB%20diagnostics%20pipeline%20needs%20and%20solutions.pdf).

This review is structured around the WHO TPPs for pulmonary TB because, despite TPPs' limitations, they remain highly influential (we do not focus on extrapulmonary, paediatric, or drug-resistant TB, for which tests are recently reviewed elsewhere [20, 21, 22, 23]). We, therefore, appraised the ability or potential of tests and technologies to fulfil TPPs and highlighted which needs are unlikely to be met by current or near-future tests and where specific criteria require updating (Tables 1, 2, graphical overview of tests and technologies by specimen type in Fig. 2). Importantly, if certain specifications are likely unmet, it does not imply a test or technology is not worth pursuing; it could be indicative of further developmental work required (sensitivity, specificity, operational aspects), selectively applying the test or technology to the correct patient- or setting-niche, or a need for a TPP criteria recalibration. We, therefore, advocate for a nuanced approach where, just because a technology does not initially appear to approach TPP criteria, enthusiasm in the test or technology should not be dampened given the current suboptimal standard-of-care available to most patients. Lastly, we highlight future research questions that need to be answered for the more promising technologies within each TPP.

## Triage Tools

Table 1 provides an overview of the triage test landscape and provides examples of different tests, their performance (including best available sensitivity and specificity estimates), and open questions [24, 25, 26, 27, 28, 29, 30]. Key themes are summarized below. Importantly, the triage and confirmatory categories are not mutually exclusive; several tests could be used interchangeably depending on context and threshold for diagnosis and treatment initiation.

### Current

#### Symptoms

Symptom screening, which does not require infrastructure, is the most widely used triage tool; however, it is inefficient and new approaches are needed that complement symptoms or substitute them completely [31]. Critical further limitations include that, in prevalence surveys, more than half of people with TB have no symptoms and the duration of TB prior to symptoms is long (6 months) and highly varied, with certain patient subtypes having subclinical disease with presumed (albeit not yet proven) infectiousness, which does not require cough [32], at least 5 years prior to symptoms [33, 34]. Furthermore, TB cases with less severe symptoms are, compared to patients with more severe symptoms, more likely to be infectious [35], further highlighting the importance of early diagnosis prior to clinical worsening to limit transmission. In terms of sensitivity and specificity in HIV-positive people, symptoms (i.e., the WHO four symptom screen) have 83% sensitivity and 38% specificity, with sensitivity lowest in outpatients on ART and pregnant women, and specificity lowest in inpatients [36]. The key weakness of syndromic screening is specificity: most patients referred for confirmatory diagnoses will be further investigated unnecessarily for TB and their true underlying healthcare needs will remain undiscovered [37]. Notably, the sensitivity and specificity of the WHO four symptom screen does not meet TPP benchmarks (concerningly, the performance is often further worsened by facilities unable to do high-quality symptom screening [31, 38, 39, 40, 41]). In other words, through our continued sole reliance on symptoms as the primary triage means, we have only been capturing patients who have advanced TB and in whom, from a clinical and transmission perspective, considerable damage has already been done. Pure syndromic-based screening is an outdated status quo.

#### C-Reactive Protein

C-reactive protein (CRP) is a biomarker elevated in response to inflammation or infection [42]. Traditionally, CRP was considered non-specific; however, with the advent of POC (and even potentially instrument-free [43]) tests and the synthesis and meta-analysis of data from well-design clinical evaluations [44], CRP has emerged as a tool superior to symptoms in high TB burden settings where specificity is high. For example, the WHO has advised that CRP (>5 mg/L) is incorporated into diagnostic algorithms in PLHIV because CRP is as sensitive (78%) and more specific (73%) than symptoms. However, given the relative recency of the WHO update, programmatic implementation is lacking, and CRP is not yet incorporated into most national algorithms. In contrast to outpatients, where other infections are likely more common (and of higher severity) like in inpatients CRP, will have decreased specificity and may not be as useful yet more data are needed. CRP's potential also requires investigation in extrapulmonary TB, where referral from primary care to a distant facility for invasive sampling (e.g., fine needle aspirate biopsy [45]) is needed, as well as investigation in HIV-negative patients, where emerging data suggest it may have utility and perform similar to in PLHIV (after threshold adjustment) [46, 47].

#### Digital CXR with Automated CAD

Digital chest X-ray (dCXR), the hardware for which is increasingly available in portable formats (e.g., FujiFILM XAIR) has recently been coupled with AI software that does CAD within minutes of image capture. Field performance data are promising, including for community-based screening [48, 49, 50]. CAD will be especially useful in resource-limited high-TB burden settings with a shortage of professional radiologists; however, implementation will be challenging in rural areas given expensive hardware and computer and internet requirements [7]. The most promising software packages include qXRv2 and CAD4TB (v6), with sensitivities >90% and specificities >70% [48, 49, 50, 51]. Crucially, the development and training (and independent validation) of these software algorithms was enhanced by large publicly available image collections. Similar open data sharing approaches are required to validate next-generation diagnostic software, tests, and biomarkers, which are increasingly reliant on machine learning for “biomarker” discovery. Questions regarding dCXR and CAD that remain open include performance in settings with large number of patients with existing chest abnormalities like previous TB (such patients are a cost-effective and epidemiologically important target for interventions [52]) and the utility of CAD for other respiratory diseases (e.g., SARS-CoV-2) in patients investigated for TB.

### Future

#### Cough Sounds

Cough is caused by a myriad of pathologies [53], but, in many settings and patient types, the most common cause of cough is TB. Using digital signal analysis, specific cough sound features obtained using a standard digital microphone from a brief forced cough session (seconds long) can determine if a patient has an increased probability of TB [54]. Such sound features may reflect semi-specific changes to the lung architecture associated with TB. Therefore, cough sound analysis (which is distinct from cough detection) has triage potential: one study found TB patients could be differentiated from symptomatic patients who subsequently had TB ruled out with 93% sensitivity and 95% specificity, exceeding TPP targets [55]. Moreover, this tool is inexpensive, can be done at POC, is non-technical, and can use user-friendly software [54]. The primary key challenge is diagnostic accuracy validation.

#### Breath

*Mtb* and the changes it induces in the body result in airborne volatile organic compounds [56] that can be used as breath-based diagnostic biomarkers [57]. One portable mass spectroscopy technology that detects host compounds (Aeonose) has 78% sensitivity and 42% specificity in symptomatic patients [58], while a rapid biosensor breathalyser has shown ∼97–99% and ∼97% sensitivity and specificity, respectively [59].

#### Aerosol

Aerosol is inherently attractive for sampling: all patients can provide aerosol (even without coughing), it is non-invasive to collect, and, as transmission occurs via the airborne route, readouts likely directly correlate with infectiousness, meaning aerosol-positive patients are likely more infectious than aerosol-negative TB cases. Specific constituents enriched in TB cases known to differentiate them from sick patients without TB scan originate from the host (e.g., in response to inflammation and infection) or be part of or produced by *Mtb* [56, 57]. However, a recent systematic review considered data from electronic-nosed-based diagnostics too heterogenous to pool (partly due to many varied different methods used) for evidence-based decision making [59]. Hardware also remains challenging for POC implementation but recent exciting advances in capture technologies (most notably, collection membranes in facemasks wearable for long periods [60] and cough tubes [61]) open new doors, especially as the collected material can be used with existing molecular testing technologies like Ultra.

#### Blood

Lateral flow tests are routinely used in many settings for HIV, which is successfully integrated within most national TB programmes [62, 63], and most clinic entrants are offered automatic upfront HIV testing. It, therefore, stands to reason that a similar fingerprick blood test would be readily implementable for TB triage (and, if sufficiently specificity, provide a confirmatory diagnosis). Extensive biomarker discovery work [64, 65] characterized host cytokine signatures with diagnostic potential [66]. Two such signatures are now in the process of being incorporated into the MultiBiomarker Test and SeroSelectTB tests [67, 68]. Importantly, these and other assays could be applied to fluids other than blood like saliva [69, 70]. On a conceptual level, a major challenge for assays such as these that are reliant on host responses is global variation in immune responses to diverse *Mtb* strains. Hence, developers may need to consider either designing setting- or population-specific tests with variations of core signatures or incorporating their tests into diagnostic algorithms that mitigate cytokine assay performance shortcomings through the use of other tests. Concerningly, there appear to be no other similar assays at this developmental stage, indicating a potential diagnostic technology pipeline gap.

## Confirmatory Tools

Table 2 provides an overview of confirmatory tests and technologies, including detailed sensitivity and specificity data [71, 72, 73]. Key considerations are below.

### Current

#### Sputum

Xpert MTB/RIF (Xpert) and its successor Ultra revolutionized diagnosis (Ultra has 88% sensitivity and 96% specificity) [74]. Small improvements in the performance (and POC suitability) of sputum-based diagnostic technologies continue [75, 76, 77, 78]. For example, GeneXpert Edge is a portable battery-powered solution operated by a tablet able to do Ultra onsite in difficult-to-reach populations [79]. This can facilitate home-based testing, which patients can prefer to facility testing [80]. Fast-follower technologies like Truenat MTB (Molbio Diagnostics, India) are emerging with similar sensitivity and specificity to Xpert [14] (and Truenat MTB's successor MTB Plus, expected to have similar performance to Ultra) that can also be used in a portable battery-operated platform and is WHO-endorsed [81, 82]. TB loop-mediated isothermal amplification (TB-LAMP; Eiken, Japan) is another WHO-endorsed with comparable sensitivity and specificity to Xpert (TB-LAMP lacks drug susceptibility testing) [83]. These Xpert and Ultra alternatives are important: a key limitation of the sputum testing landscape is overreliance on a single manufacturer (Cepheid), especially considering recent COVID-19-related supply chain disruptions. Programmes need more options to make decentralized molecular testing flexible and feasible and some other promising NAATs are Q-POC (QuantuMDx), IRON-qPCR (Bioneer), and NanoDetector (Ontera) [84]; however, there are limited performance data, especially outside centralized laboratories.

Importantly, NAATs reduced demand for mycobacterial culture, which requires specialized infrastructure; however, it is worth noting that culture is still useful if patients are NAAT-negative and clinical suspicion remains, or if there is a high probability of NAAT false-positivity (e.g., in recently previously treated patients [77, 85, 86]). Importantly, in the latter scenario, novel *Mtb* RNA-based assays may have further discriminatory power [87]. One important research question is how frequently, if at all, programmatically generated diagnostic culture results are actually used for clinical decision making and if this justifies the time (including delay) and expense involved [88]. Continuing to reduce the need for culture will likely prove fruitful for clinicians and programmes; however, it will continue to have an increasingly niche role as confirmatory test.

Fundamentally, however, the next TB diagnostic game-changer is unlikely to come from any sputum tests: limits-of-detection are already excellent (approximating 10–100 CFU/mL), leaving little room for improvement. Sputum is therefore arguably a specimen variety that has, for the purpose of initial diagnosis, already attained close to its maximum potential. Furthermore, given the implementation fatigue associated with the roll-out of the GeneXpert technologies, programmes are likely only willing to embark on the huge effort required to introduce a new confirmatory test if it represents a revolutionary paradigm shift (i.e., is sputum free) rather than a small incremental increase in sensitivity and specificity using a specimen such as sputum with well-known limitations.

#### Urine

Urine is easy to collect and poses less infectious risk than sputum. Furthermore, urinary approaches are inherently complementary to traditional sputum-based testing. For example, sick patients with paucibacillary sputum (e.g., PLHIV) or unable to produce sputum (e.g., inpatients) are more likely to have advanced TB disease (which could be extrapulmonary). Furthermore, HIV-associated renal dysfunction is common in such patients; this can increase urinary biomarker levels [89]. Urine is also the only non-sputum specimen for which a WHO-endorsed confirmatory test already exists. AlereLAM has 42% sensitivity and 91% specificity in PLHIV [90], meets the WHO operational TPP criteria for a biomarker-based non-sputum test, and has robust evidence showing a clear impact on long-term patient health. Concerningly, however, AlereLAM's uptake has been disappointingly slow: TB programmes consider it a “niche test” and there has been a coordination between HIV and TB programmes within countries [91]. Users of next-generation tests (some of which are detailed later) should be aware of these lessons learnt from the AlereLAM experience. Finally, Ultra has been applied to urine for both pulmonary and extrapulmonary TB diagnosis; however, yields are generally low [45, 92] albeit very ill HIV-positive inpatients are a possible exception [93].

#### Stool

Stool diagnostics detect *Mtb* that likely originates from swallowed sputum. Although it is arguably not as readily accessible as blood and urine (rectal swabs may be an alternative), stool may be better compared to the other invasive sampling strategies like aspirates or bronchoscopy in individuals who struggle to provide a good quality specimen. Stool testing using Ultra is WHO-endorsed in children [94] with 61% sensitivity and high specificity (>98%); however, data from adults are very limited [95]. A key challenge facing future stool-based tests is specimen processing. Solutions like the Simple One-Step Stool Method adds no additional cost prior to Xpert testing as it uses the same buffer [94] but more improvements are needed. Lastly, it is unclear − both for adults and children − how stool testing should be incorporated into national diagnostic algorithms.

### Future

#### Tongue Swabs

Respiratory secretions passing in the oral cavity adhere to tongue papillae, permitting *Mtb* to concentrate in tongue dorsum biofilms. These biofilms are easily accessible, can be self-sampled, and the material collected is inputted into existing test platforms. For example, Ultra on a single tongue swab had a sensitivity of 88% in symptomatic outpatients [96], whereas another study in patients with less advanced disease (prison-based active case finding) showed 43% sensitivity [97]. It should be emphasized, however, that Ultra's specimen processing steps are designed for sputum, not tongue swabs, and that approaches (or tests) optimized to maximize *Mtb* DNA yield from tongue swabs may enhance this promising approach's sensitivity. Importantly, part of this is ensuring that maximum amount of biomass is captured by the swab and as much as possible is released for diagnostic detection.

#### Blood

Upcoming tests based on host transcriptomic signatures had considerable promise [98, 99]; however, independent validations have shown worse than expected performances [100, 101]. Nevertheless, the likely first-to-market test in this category is MTB-HR. When evaluated as a confirmatory test in a multicentre study, it had showed 90% sensitivity and 86% specificity [19] (other studies have reported significantly lower specificities [102, 103]); however, head-to-head performance data using other promising blood biomarkers like CRP are absent from these evaluations and should be routinely included as a comparator. Another design locked is TAM-TB that measures T-cell activation in response to TB and has demonstrated potential to meet TPP targets [104, 105]; however, as TAM-TB is a flow cytometry-based assay, it has limited POC potential. Yet, together with MTB-HR, it is one of the most promising non-sputum confirmatory test candidates.

#### Urine

Most upcoming urine tests target LAM or *Mtb* DNA. SILVAMP TB LAM (SILVAMP; Fujifilm, Japan) will likely be the first to market next-generation LAM test after AlereLAM, over which it has 28% improved sensitivity in HIV-positive inpatients at high specificity [106]. Most excitingly, SILVAMP and its successors should make LAM testing feasible in all at-risk patients irrespective of HIV status due to higher sensitivity in HIV-negatives (53% SILVAMP vs. 11% AlereLAM). The damaging notation that the biomarker LAM is therefore inherently useful only in a minority of patients can thus be dispelled. Another form of promising urine-based tests, albeit at an earlier development stage, is cell-free DNA detection. These are *Mtb* fragments released into the blood (where they can also be detected) and subsequently excreted in urine [107, 108]; however, no commercial products exist.

### A Potential Role for Multi-Pathogen Diagnostic Panels?

Only 10–20% of patients presenting to TB clinics with cough have TB, and, for most patients in high burden settings, their diagnostic journey stops prematurely when a negative TB result is returned. Many different respiratory pathogens can now be detected in multiplex panels; however, this approach is relatively unexplored for TB. Such panels would also permit data to be generated on potential non-TB causes of disease. Several multi-pathogen diagnostic panels like FilmArray Respiratory Panel (Biofire Diagnostics), the Respiratory Multi Well System MWS r-gene Range (BioMerieux), FTD 21, and FTD 33 respiratory panels (FTD), Respiratory Pathogen ID/AMR Panel (Illumina) are potential candidate technologies; however, further development and research is warranted to simplify these tests and make them more useful for the types of populations in which investigations for TB are frequent [109].

### How Can We Apply Promising New Technologies?

Strategies reliant on passive diagnosis in self-presenting patients recognized as symptomatic by under-resourced facilities will never be enough to curtail transmission. Active case finding, including for subclinical TB in communities is needed. Furthermore, introducing up-to-date information technology infrastructure that provides diagnostic connectivity solutions, already strengthened globally due to COVID-19 [110], and automated result reading should accompany the development of new technologies so as to reduce delays inherent in paper-based systems [111] and permit better data sharing across programmes to improve linkage to care. Of the new triage technologies, CRP and CAD with dCXR are increasingly feasible in resource-limited settings and their implementation should proceed in combination with existing confirmatory tests like Truenat, which could be done near POC (using GeneXpert Edge, for example). Eventually, however, confirmatory testing should be sputum-free; however, the lack of non-sputum confirmatory tests should not halt deployment of our best available triage methods (where diminished specificity is more tolerable). Different combinations of tests used as part of a holistic algorithm need to be modeled to determine the number needed to test to identify (or exclude) TB and whether this meets willingness-to-accept thresholds. Importantly, these decisions will be dependent on the setting and patient population, and models require input data from diverse settings. Overall, an agile approach to avoid a “one size fits none” situation needs to be taken, and local decision making should be facilitated by policymakers and national programmes. Furthermore, one hitherto largely unappreciated aspect of these new technologies (e.g., dCXR with CAD) is they could provide valuable information for the diagnosis of diseases that mimic TB. Revisiting existing diagnostic algorithms and factoring in these “non-TB” benefits for technologies like CAD will help drive adoption of novel (yet costly) tests.

## Conclusion

There are several promising diagnostic technologies, and the onus is on policymakers, researchers, and health workers to ensure that their potential is capitalized upon in the context of their inherent limitations. New diagnostic performance data are essential to generate for review by the WHO, especially for non-sputum-based tests, and this should be done using revised and updated TPP criteria. Furthermore, new tests are needed where pipeline gaps exist and late-stage candidates are few (e.g., design-locked fingerprick blood tests, non-sputum confirmatory tests). To promote POC testing, innovation is needed to develop “agnostic” technologies that could benefit a variety of different tests types. These technologies could include methods of specimen or biomarker concentration not exclusive to a specific test but, in principle, are widely deployable within different assays. Furthermore, while sensitivity and specificity are important, equal focus on important aspects essential for POC deployment like power supply and user friendliness is required. The time to target patients beyond just those with symptoms already at facilities is more feasible and compelling than ever, and the speed at which excellent COVID-19 tests were developed puts into greater perspective the inertia in not only new TB test development but also the lagging deployment of already existing tools that could save the lives of people suffering from TB now (e.g., CRP, dCXR with CAD).

## Conflict of Interest Statement

Grant Therons's research group has received funding and/or in-kind donations in the last 5 years via his employer Stellenbosch University from Bruker Hain Lifesciences, Cepheid, LumiraDx, FIND, Biopromic, Newmark Diagnostics, HemoCue, Boditech, and Copan. Byron W.P. Reeve received travel support from Cepheid to attend a conference and present unrelated data. Coenraad F.N. Koegelenberg is an Associate Editor of “Respiration”. The authors have no financial involvement with any organization or entity with a financial interest in, or financial conflict with, the subject matter or materials discussed in the manuscript apart from those disclosed.

## Funding Sources

Grant Theron acknowledges funding from South African Medical Research Council and the EDCTP2 programme supported by the European Union (SF1401, OPTIMAL DIAGNOSIS; RIA2020I-3305, CAGE-TB) and the National Institutes of Health (U01AI152087; U54EB027049; D43TW010350). Shima M. Abdulgader, Anna O. Okunola, and Byron W.P. Reeve acknowledge receipt of funding from EDCTP. Anna O. Okunola acknowledges funding from the EDCTP2 programme supported by the European Union (TMA2020CDF-3209-RADIANT). Gcobisa Ndlangalavu acknowledges funding from the SAMRC. The work reported herein was made possible through funding by the South African Medical Research Council through its Division of Research Capacity Development under the Internship Scholarship Programme from funding received from the South African National Treasury. The content hereof is the sole responsibility of the authors and does not necessarily represent the official views of the SAMRC or the funders. Grant Theron confirms he had final responsibility for the decision to submit for publication.

## Author Contributions

Grant Theron conceptualized the review. Shima M. Abdulgader, Anna O. Okunola, and Gcobisa Ndlangalavu wrote the first draft. Shima M. Abdulgader, Anna O. Okunola, Gcobisa Ndlangalavu, Byron W.P. Reeve, Brian W. Allwood, Coenraad F.N. Koegelenberg, Rob M. Warren, and Grant Theron critiqued and edited the manuscript.

## Figures and Tables

**Table 1:** Overview of selected current and future *triage* tests, how they work, evidence to support use, key challenges, and opportunities, and recommendations for clinical and programmatic decision makers

Triage test	How it works	Evidence for use with examples of (potential) products	Challenges and opportunities	Recommendations and future work
*Clinical examinations and imaging*				

Symptom screen	Health workers screen patients using a questionnaire.	Consumable-free, cheap, rapid, and doable at POC. WHO symptom screen has a sensitivity and specificity of 70% and 50%, respectively [24]. Subjective and vulnerable to self-reporting biases and poor quality-of-care [25,41]3.	Misses cases that do not report symptoms. Does not meet TPP criteria. Patients who screenpositive by symptoms yet are confirmatory test-negative are generally not further investigated, representing a missed opportunity. New clinical prediction rules may offer superior performance [26], including when combined with routine biomarkers readouts (e.g., Hb) but these require evaluation.	Symptoms should not be used to rule-out TB. Likely significant operational change needed to reduce health programmes' reliance on symptoms. Sufficient evidence exists for PLHIV to move away from a sole reliance on symptoms. Future role of symptoms (if any) alone or with other tests requires clarification.

dCXR	dCXRs can be collected using a low-dose portable unit close to near POC with minimal infrastructure requirements. Images are analysed locally or online.	Commercial hardware solutions include Al-equipped Delft Light, FDR Xair, Impact, and HandMed units [84]. qXRv2, CAD4TB, and Insight CXR are examples of software, with sensitivities of 95% each, and specificities of 82%, 80%, 76%, respectively [48–51].	Requires expensive hardware but infrastructure and costs requirements diminishing. Cost per patient small after instrumentation outlay. CAD solutions can be used with existing image capture platforms; however, these are usually centralized and not POC in high burden countries.	Recommendations regarding clinical action post-CAD requires incorporation into programmatic algorithms and these should factor in CADs sensitivity for asymptomatic TB [27]. If CAD software returns a negative TB result, software for other diseases could be run; however, research is needed before this is considered for implementation.

*Blood*				

CRP	Elevated CRP implies inflammation or infection, which may be TB. New platforms permit accurate CRP measurement in fingerprick blood at POC.	Sensitivity and specificity of 78% and 73%, respectively, in PLHIV (>5 mg/L) [28]. CRP can be tested using near POC platforms such as Boditech ichroma II [28] or LumiraDx [29].	No lateral flow assay formats for true POC testing.	More data in HIV-negatives and EPTB is needed. Utility in combination with other tests requires assessment − CRP should always be included as a comparator in studies. CRP likely useful for more than just TB: elevated CRP in TB-negatives should inform diagnosis of other diseases.

Host transcriptomics	Levels of specific host RNA signatures in fingerprick blood are elevated in TB and measurable by techniques like RT-PCR.	Many signatures exist [98], and, in a head-to-head validation, Sweeney3 (a TB signature consist of the 3 transcripts GBP5, DUSP3, KLF2) had the highest AUROC point estimate [100]. Sweeney3 is incorporated into what will likely be the first-to-market host RNA commercial TB test: MTB-HR. MTB-HR had 90% sensitivity and 86% specificity in a multicentre study [19], with other studies reporting specificities of 53–56% [102, 103].	More complex signatures (>3 targets) are hard to integrate in POC platforms. Limited validation data, including in exposed contacts for incident TB.	Host RNA is labile, and operationalizing tests to detect this will be challenging and expensive. DBSs need investigation. RNA signatures appear unlikely to meet optimum TPP criteria and come close to approaching minimum criteria in high-burden settings only [99]. Further research is required across the spectrum of the TB disease.

Cytokines	Tests in lateral flow assay formats, like SeroSelectTB (immobilized combinations of high affinity TB antigens) and MBT (exact target unknown), detect host immune markers associated with TB on non-stimulated blood.	SeroSelectTB's [68] accuracy for TB and its effect on patient outcomes is undergoing evaluation in South Africa, Ethiopia, and Tanzania. MBT is undergoing evaluation in South Africa, The Gambia, and Uganda [30].	Both assays are currently commercially unavailable and not WHO-endorsed. More late-stage candidate assays are needed.	More evaluations needed in populations other than of adults with presumptive pulmonary TB, including children, EPTB, and asymptomatic outpatients.

*Sounds and breath*				

Cough or chest sounds	Automated analysis of sounds captured using a standard digital microphone, like that of a smartphone, can differentiate coughs from people with TB and people without TB.	Proof-of-concept work shows that sounds can distinguish TB from sick non-TB patients with 95% sensitivity and 72% specificity [54].	Identifying a universal cough audio triage signature for TB may be unlikely given regional differences, hence individual settings (and potentially different types of patients) may require different signatures to be measured. This approach otherwise has great potential, as it is specimen-free and uses existing smartphones.	Large collections of sounds from well-characterized patients require analysis using a “train and test model”, like that used for Al-based dCXR CAD approaches.

Breath and aerosol	Collected breath, for example, in EBCs, can be analysed by mass spectroscopy to identify metabolic compounds including VOCs that correlate with TB status. Another component of breath is aerosol, which contains *Mtb* cells or components like DNA, which may be detectable using currently available assays designed for sputum (e.g., Ultra).	VOC-based electronic noses have 93% sensitivity and 93% specificity (pooled estimates from an meta-analysis) [59]; however, this estimate is comprised of different products. Aeonose, perhaps the most researched breath-based test has 81% sensitivity and 60% specificity [84].	Sampling in rapid and specimen collection should be readily deployable at scale (detected at POC may remain a challenge). Aeonose does not meet the TPP sensitivity and specificity community triage requirements.	Diagnostic studies at a large scale, including in active case finding scenarios, are needed to validate this promising technology.

Tests in the triage category could, under certain circumstances, serve as confirmatory tests and vice versa. Al, artificial intelligence; ART, antiretroviral therapy; AUROC, area under receiver operator curve; CAD, computer-aided detection; CRP, C-reactive protein; EBCs, exhaled breath condensate; EPTB, extrapulmonary TB; DBS, dry blood spots; dCXR, digital chest X-ray; RT-PCR, real-time polymerase chain reaction; POC, point-of-care; TPP, target product profile; TB, tuberculosis; MBT, multibiomarker test; MTB-HR, Xpert MTB/Host Response; VOC, volatile organic compounds; WHO, World Health Organization.

**Table 2:** Overview of selected current and future *confirmatory* tests with evidence to support use, key challenges, and opportunities, and recommendations for clinical and programmatic decision makers

Confirmatory test	How it works	Evidence for use with examples of (potential) products	Challenges and opportunities	Recommendations and future work
Sputum				

Ultra	Ultra detects *Mtb* DNA in sputum specimens by targeting multi-and single-copy loci. Ultra uses the widely deployed GeneXpert system. Key strengths are the mostly automated specimen processing and DNA extraction steps, a feature that few other products have, and the ability to use the GeneXpert platform for other test types.	Ultra has 90% sensitivity and 96% specificity [71]; however, data in patients with less advanced forms of disease and/or HIV (whom are more likely to have paucibacillary sputum) are under-represented.	Expensive, requires constant electricity and controlled environment. Xpert Edge is a hardware solution that can run GeneXpert cartridges, including Ultra, and may improve decentralizing. Ultra is sometimes prone to positive results in culture-negative patients. Their clinical management clarification, especially in the context of community case finding.	More evaluations in groups other than patient with typical presumptive pulmonary TB needed. Implementors need to strengthen guidance for health workers regarding “trace” semi-quantitation category result handling to prevent diminished confidence in Ultra. GeneXpert needs to be optimized.

Truenat	Truenat MTB and Truenat MTB Plus are portable NAATs that target single-copy and/or multi-copy loci.	Truenat MTB and Truenat MTB Plus had a pooled sensitivity of 73% and 80%, respectively, approximating Xpert in the same study [14]. Among smear-negative specimens, sensitivities were 36% and 47%, respectively.	Portable battery-powered platform Test is not automated to the degree of GeneXpert (requires pipetting between stages).	Wider clinical evaluations outside of India are needed, especially where HIV, previous TB, and drug resistance are common. Improvements to meet optimum TPP targets are needed.

*Blood*				

Xpert MTB-HR	This qRT-PCR quantifies relative host mRNA levels of the 3-gene GBP5, DUSP3, and KLF2 signature. Cartridges can use existing GeneXpert machines in facilities that Ultra.	A recent multicentre study showed 90% sensitivity and 86% specificity [19].	Without specimen preservatives, which add expense, the assay needs to be done within 30 min of blood collection, which may not be feasible unless POC facilities are available.	Evaluation studies to be conducted across diverse populations to define signature cut-offs for active and incipient TB. Larger multicentre evaluations needed to monitor at-risk groups potentially transitioning across the spectrum of disease from exposure to active TB.

TAM-TB	T-cell activation marker patterns measured by flow cytometry are used to discern active TB.	A study reported the assay, on 1 mL of blood, to have 82% sensitivity and 93% specificity in presumptive TB patients [105].	Giving instrumentation requirements, this assay will likely initially be based at centralized laboratories; however, this is not a critical caveat given TAM-TB could deliver sputum-free confirmatory diagnoses. Performance appears unaffected by HIV, which is a major strength [105].	Independent validation studies are required, including in special patient groups and from diverse regions. Development work to reduce the hardware requirements and improve POC placement highly desired.

*Urine*				

LAM	LAM is a component of the *Mtb* cell wall present in patients' urine. New-generation tests include improvements in antibody affinity, to specimen processing and signal detection, enhancing limits of detection.	Of the next generation assays, FujiLAM has the most data to support use, with 35–40% greater sensitivity among both HIV-positive and -negative patients compared to AlereLAM (FujiFILM's overall sensitivity and specificity were 77% and 92%, respectively). Other new LAM assays but with more limited performance data include FLOW-TB (Salus Dsicovery), EcLAM (Meso Scale Diagnostics, and assays by Biopromic and Mologic.	FujiLAM meets TPP minimum sensitivity and specificity criteria for a non-sputum TB test. FujiLAM does not need additional infrastructure and can be applied at POC. New assays that require instrumentation will be disadvantaged. A unique opportunity exists to expand urine LAM testing to HIV-negatives in a feasible manner.	Careful implementation of new LAM assays is required to ensure they are not underutilized to the extent AlereLAM is. Evaluation outside of typical patient groups with pulmonary symptoms is required. How diagnostic algorithms could optimally incorporate new LAM tests in concert with other diagnostics requires clarification.

cfDNA	Short (<100bp) extracellular *Mtb* DNA fragments circulating in bodily fluids and may be detectable using NAATs. This approach can also be applied to blood.	An early phase retrospective validation study of a urine cfDNA assay that detects <5 copies of cfDNA had 83% sensitivity and 100% specificity [107]. In blood-based cfDNA detection sensitivity ranged between 43 and 65% and specificity between 67 and 93% [72].	cfDNA is a small molecule and the variation in the specimen type, collection method, and isolation protocol affect the performance [73]. The assay is highly technical; therefore, it is not suitable for POC testing in the current format, but cartridge-based assays are in development.	Improvements in cfDNA capturing techniques may increase sensitivities. Instrument-free POC platforms may prove challenging given the DNA capture and detection requires. Larger diagnostic studies are needed to confirm the preliminary data. These should use semi-automated rather than manual PCR methods.

*Oral*				

Tongue swabs	Tongue papillae filter respiratory secretions, trapping bacilli, and acting as a *Mtb* cell concentrator over long periods.	Relative to sputum Ultra, the sensitivity was 88% for a single swab, while specificity was 79% [96].	Initial data were generated using in-house PCR methods and future studies should combine tongue swabs with existing commercial platforms. Patients could self-swab at POC, and swabs be sent to a central facility.	Additional work required on optimizing DNA liberation from swabs. Evaluations in patient groups other than standard presumptive pulmonary TB are required, including in sputum-scarce patients.

cfDNA, cell-free DNA; LAM, lipoarabinomannan; *Mtb,* mycobacterium tuberculosis; NAAT, nucleic acid amplification test; qRT-PCR, quantitative real-time polymerase chain reaction; ΤΑΜ, T-cell activation marker; POC, point-of-care; TPP, target product profile; TB, tuberculosis.
